# Effect of the Carbon-Curved Cane Use on Gait in Chronic Stroke–Induced Hemiplegia: A Prospective Single-Case Study

**DOI:** 10.1155/carm/7294729

**Published:** 2025-03-26

**Authors:** Ryu Kokuwa, Yuta Sakai, Yosuke Nagasaka, Yuki Iwama, Kazune Matsubara, Yuuma Ueno, Seiichi Matsushita, Junpei Ishikawa

**Affiliations:** ^1^Syupoon Inc., 43, Nishimaeshinden, Uguiura-Cho, Yatomi, Aichi 498-0026, Japan; ^2^Department of Paracane, Welloop Inc., 43, Nishimaeshinden, Uguiura-Cho, Yatomi, Aichi 498-0026, Japan; ^3^Nagoya Municipal Industrial Research Institute, 3-4-41, Rokuban, Atsuta, Nagoya, Aichi, Japan

## Abstract

**Background:** Canes are used by patients with hemiplegia to improve gait and ambulation, but the effects of different types of canes remain unclear. Therefore, this study compared the effectiveness of a newly developed carbon-curved cane (CC-C) with that of a conventional cane using gait analysis of patients with chronic stroke–induced hemiplegia.

**Case:** A 41-year-old male was diagnosed with cardiogenic cerebral infarction 3 years ago. The patient is independent in his activities of daily living and participates alone in the community using a single-point cane (SP-C). This study utilized an AB design with two conditions: the use of an SP-C and a CC-C. Gait evaluation included a three-dimensional gait analysis and analysis of the ground reaction force (GRF) applied to the cane using a force plate. The main outcomes were the spatiotemporal gait characteristics, and the suboutcomes were range of motion (ROM), center of mass (COM) trajectory, and GRF applied to the cane. Wilcoxon rank-sum test was performed to clarify the difference between SP-C and CC-C use with a significance level of *p*=0.05. Gait velocity, paretic and nonparetic step length, stride length, cadence, and single-stance time tended to be higher, and the preswing time was lower with CC-C than with SP-C use (*p* < 0.05). Differences were observed in limb ROM and COM trajectory (*p* < 0.05) with GRF tending to have a higher propulsion force in CC-C and SP-C having higher braking and medial forces.

**Conclusion:** CC-C improved gait and demonstrated different GRF values than SP-C.

## 1. Introduction

Patients with stroke-induced hemiplegia exhibit more abnormal gait patterns than healthy people [1, 2]. These abnormal patterns are observed not only in the paretic lower limb but also in the upper limb, trunk, and nonparetic lower limb [3–5]. These patients also have reduced speed [6], poor gait efficiency [7], and increased metabolic cost [8] compared to healthy people. Considering that gait velocity is a factor that determines the hierarchy of the range of life with stroke-induced hemiplegia [9] and that gait independence influences whether patients can return to their homes, gait ability is an important factor for patients with stroke-induced hemiplegia. Therefore, improvement in gait ability is a major goal of rehabilitation aimed at maximizing independence in activities of daily living (ADL) at home and in the community.

Gait reconstruction requires exercise therapy and the use of effective assistive devices, such as canes, lower limb orthoses, and upper limb assistive devices [10]. Therefore, assistive devices are used in gait rehabilitation and chronically in homes and communities. Kuan, Tsou, and Su [11] compared patients with stroke-induced hemiplegia with and without the use of a cane and demonstrated an increased gait velocity and step length of the paretic lower limb and reduced circumduction during the swing phase. This was because the cane facilitated the shift of the center of mass (COM) to the nonparetic side and assisted the push-off of the paretic lower limb. Polese et al. [12] reported an increase in lower limb muscle activity during the stance phase of walking when using a cane. Thus, cane usage contributes to gait reconstruction in patients with stroke-induced hemiplegia during both the stance and swing phases of gait.

Canes are classified as single-point canes (SP-Cs) and multiple-point canes (MP-Cs). SP-C is primarily used in mild cases, whereas MP-C is utilized in severe cases. Jeong et al. [13] reported that individuals with hemiplegia who have relatively poor balance skills (i.e., Berg Balance Scale < 49) were able to walk with a four-point cane with less oxygen consumption than when using a single cane. Contrastingly, Beauchamp et al. [14] reported that the asymmetric gait pattern in mild stroke-induced hemiplegia was better using an SP-C than a four-point cane. These results indicate that using an appropriate cane according to the patient's gait ability can have desirable effects. On the other hand, despite the wide variety of patient gait patterns, the types of canes are limited. Even for the typical single cane and four-point cane, no significant changes in their shapes have been observed over the years. In addition, in reports comparing different types of canes, none have examined the force components (e.g., propulsion or braking force) that affect gait. Therefore, in this study, the researchers developed a carbon-curved cane (CC-C), focusing on the flexibility and resilience of carbon, considering that it can be used during walking to further reconstruct gait. This case study aimed to verify whether the use of the newly developed CC-C grounded at the surface influences efficacy (gait performance) and usability better than the conventional SP-C.

## 2. Case Presentation

### 2.1. Participant

A 41-year-old male was diagnosed with cardiogenic cerebral infarction 3 years ago. After approximately 6 months of inpatient rehabilitation, he was discharged home and was able to perform ADL independently at home (using an SP-C or handrails) and participated in local communities (using an SP-C). After discharge, rehabilitation sessions were conducted twice weekly using community services. The patient's current performance levels are presented in Table 1.

### 2.2. CC-C

**Figure 1:**
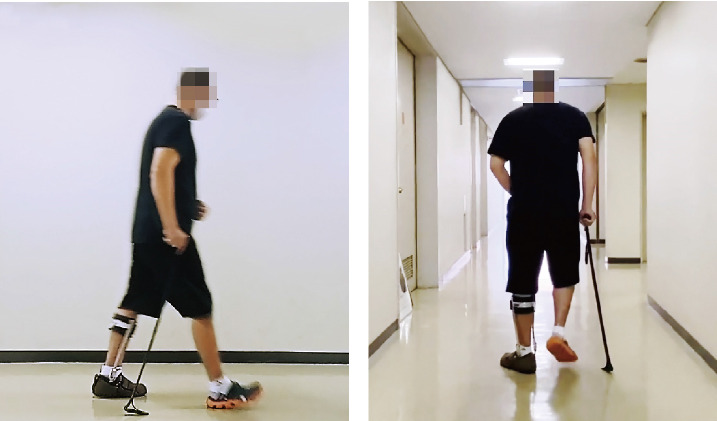
Gait with a carbon-curved cane: (a) the sagittal plane and (b) the frontal plane.

The CC-C used was a Paracane-Three Part model (Three-Part cane, Syupoon Inc., Aichi, Japan). A three-part cane made of dry carbon was shaped to make ground contact (Figure 1). This cane consists of a grip, shaft, and ground-contact portion. The main feature is that the carbon in the shaft and ground-contact portion is deflected under load and exhibits the resilient characteristic of carbon when released. The ground-contact portion was designed as a surface-type mechanism to stabilize the mechanical component added by shaft flexing.

### 2.3. Intervention

This study utilized an AB design with two conditions: the use of an SP-C (A) and a CC-C (B). The main outcomes were spatiotemporal gait characteristics determined using three-dimensional gait analysis, and the suboutcomes were range of motion (ROM), COM trajectory, and ground reaction force (GRF) applied to the cane; balance test: 10 m maximum walking speed test; timed up and go (TUG) test; and usability evaluation: System Usability Scale (SUS) and Quebec User Evaluation of Satisfaction with Assistive Technology (QUEST). To assess neurological functions and the current ability to perform ADL, the Brunnstrom Recovery Scale (BRS), Stroke Impairment Assessment Set (SIAS), Functional Independence Measure (FIM), and Mini-Mental State Examination-Japanese (MMSE-J) tests were conducted. We used the Japanese author certified version of the MMSE, which we purchased from the publisher (Nihon Bunka Kagakusha, Tokyo, Japan).

Three-dimensional gait analysis was performed using a three-dimensional motion analyzer (VENUS3D, Nobby Tech. Ltd., Tokyo, Japan) and force plate (TFP-404011B, Technology Service, Ltd., Nagano, Japan). To ensure accurate measurements, the participant was fitted with a measurement suit designed to be worn over clothing. In accordance with previous studies [4, 15], 27 markers were placed on the following anatomical landmarks: the seventh cervical vertebra, two locations on the thorax, bilateral acromion, bilateral elbow joint, bilateral ulnar process, bilateral two dorsal surfaces of the hand, bilateral iliac crests (the height of each iliac crest on the vertical line passing through the hip joint), bilateral hip joints (at one-third of the distance on the line connecting the greater trochanter and the superior anterior iliac spine), bilateral knee joints (midpoint of the anteroposterior diameter of the lateral femoral epicondyle), bilateral ankle joints (external ankle condyle), bilateral lateral lower ankle joint, bilateral second toe, and bilateral fifth metatarsal heads. The sampling frequency was set to 100 Hz for VENUS3D and 1000 Hz for the force plate. Three-dimensional gait analysis was performed on a 5 m leveled indoor straight walking path. A force plate was placed in the middle of the walking path, and the participant was required to contact the cane on the force plate once while walking comfortably. The starting position was adjusted by measuring the stride length such that the cane could be grounded during natural walking. Ten measurements were recorded under each condition, and data from one gait cycle before and after force plate contact were used (data of a total of 10 gait cycles). As this was the first time a CC-C was used in this case, the patient was provided with a thorough explanation of the function of the cane and an opportunity to practice before the measurement (approximately 20 min).

The 10 m maximum walking test was conducted indoors with a 3 m back-and-forth preliminary path [16]. The participant walked in the shortest possible duration, and the time between 10 m distances was measured using a stopwatch. Two measurements were performed for each condition, and the average value was used as the representative value. To prevent the participant from falling, the measurer walked closely alongside. It has been widely used in rehabilitation settings and has demonstrated reliability [17].

The TUG test was conducted indoors in a leveled environment, using a chair and colored cones. The participant stood up from the chair on cue, started walking, walked around a color cone that was 3 m away, and then sat down on the chair again [18]. The measurements were recorded once for rightward walking and once for leftward walking, and the average was used as a representative value. It has been reported to be a reliable indicator for evaluating balance in people with chronic stroke [19].

SUS and QUEST were administered using questionnaire tables; SUS is applied for the clinical evaluation of assistive devices [20]. The participant was asked to rate the usability of the assistive device on a 5-point scale (1 (do not agree) to 5 (agree)) for 10 questions related to usability. Each item was scored, and an overall score between 0 and 100 points was calculated. A score > 50 indicated that a user considers the system to be of “decent” usability; ≥ 70, “good” usability; > 85, “excellent” usability. The QUEST is a 12-item satisfaction index for welfare equipment and services (eight items for welfare equipment and four items for services). The participant rated each item on a 5-point scale (1 (not satisfied) to 5 (very satisfied)), and the average value was calculated for each major item [21]. This evaluation method has been reported to be reliable and valid [22, 23] and has been clinically applied to newly developed devices and welfare equipment.

### 2.4. Statistical Analysis

Spatiotemporal gait characteristics, ROM, COM trajectory, and GRF applied to a cane were compared under SP-C and CC-C conditions. After conducting the Shapiro–Wilk test for normality, the data were analyzed using the Wilcoxon rank-sum test. Statistical analysis was performed using *R* 4.0.2 (University of Auckland, Auckland, New Zealand), and statistical significance was set at *p* < 0.05.

### 2.5. Findings

The BRS, SIAS, FIM, and MMSE scores are shown in Table 1; the total SIAS and FIM scores were 37 and 116 points, respectively, and the MMSE score was 30 points.

Table 2 presents spatiotemporal gait characteristics. Overall, significant differences were observed between SP-C and CC-C conditions in gait velocity, paretic step length, nonparetic step length, stride length, cadence, single-stance time, and preswing time. Temporal parameters showed that the CC-C condition tended to last longer in single stance and the SP-C condition tended to last longer in preswing.

Table 3 shows the maximum ROM during walking and the COM trajectory. In terms of ROM, significant differences were found in the shoulder, elbow, wrist, hip, and knee joints on the paretic side; wrist motion on the nonparetic side; and trunk motion. In the upper limb on the paretic side, the elbow-extension angle tended to be greater in the CC-C condition, whereas shoulder flexion, extension angle, and elbow-flexion angle tended to be greater in the SP-C condition. The extension angle of the wrist tended to be greater in the SP-C condition in the nonparetic upper extremity. In the paretic leg, the hip-flexion, extension, and knee-flexion angles tended to be greater in the CC-C condition, whereas the knee-extension angle tended to be greater in the SP-C condition. During trunk movement, SP-C use resulted in a greater tilt to the right side and CC-C use resulted in a greater tilt to the left side. In the COM trajectory, the amount of lateral movement in the swing phase and the amount of vertical and lateral movements in the single-stance phase tended to be greater in the CC-C condition.

**Figure 2:**
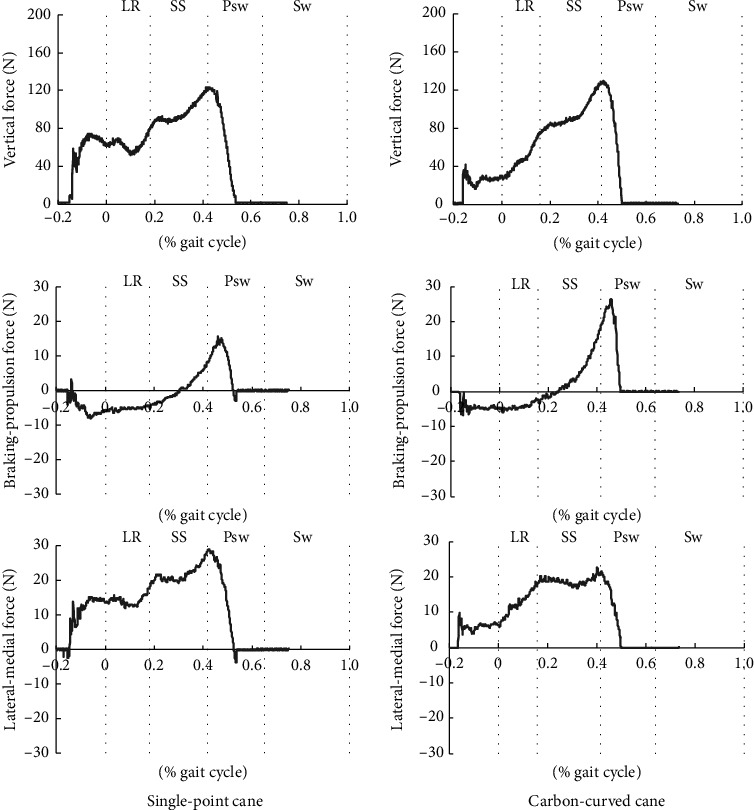
Ground reaction force applied to the cane during gait. The three leftmost graphs show the single-point cane condition, and the three rightmost graphs show the carbon-curved cane condition. The vertical axis represents the vertical force (N) in the top graph, the braking-propulsion force (N: positive value of propulsion force) in the middle graph, and the lateral-medial force (N: positive value of medial force) in the bottom graph. The horizontal axis represents the gait cycle (% gait cycle). The bold line at the center of the graph shows the average values for each condition (10 gait cycles), and the thin vertical line represents the average values when the walking cycle switches. The interval between the thin vertical lines represents each gait cycle (LR, loading response; SS, single-stance; Psw, preswing; Sw, swing).

Figure 2 shows the GRF values applied to the cane during walking. The peak force (N) and impulse (area between a component curve and the baseline: N·s) of the vertical component were 124.4 ± 7.0 N and 56.3 ± 3.7 N s in the SP-C condition and 126.9 ± 4.2 N and 54.5 ± 2.8 N s in the CC-C condition (mean ± standard deviation (SD)), respectively, with no significant differences between conditions (effect size: 0.67 and 0.20, respectively). The peak force and impulse of the braking component were 9.6 ± 1.5 N and 3.0 ± 0.7 N s in the SP-C condition and 8.2 ± 1.1 N and 2.5 ± 0.7 N s in the CC-C condition, respectively. The peak force and impulse tended to be significantly larger in the SP-C condition (effect size: 0.01 and 0.03, respectively). The peak force and impulse of the propulsion component were 17.8 ± 2.7 N and 1.5 ± 0.3 N s in the SP-C condition and 29.2 ± 1.6 N and 2.4 ± 0.4 N s in the CC-C condition, respectively. The peak force and impulse tended to be significantly larger in the CC-C condition (effect size: 0.00 and 0.00, respectively). The peak force and impulse of the medial component were 29.9 ± 1.9 N and 10.5 ± 1.8 N s in the SP-C condition and 22.1 ± 2.2 N and 7.2 ± 1.1 N s in the CC-C condition, respectively. The peak force and impulse tended to be significantly larger in the SP-C condition (effect size: 0.00 and 0.00, respectively).

Table 4 presents the balance test and usability scale scores. The 10 m maximum walking test and TUG test results were 7.25 s and 12.43 s for the SP-C condition and 6.82 s and 10.58 s for the CC-C condition. The overall SUS and QUEST scores were 45 and 2.50 points for the SP-C condition and 95 and 4.00 points for the CC-C condition, respectively.

## 3. Discussion

In this study, to verify the effectiveness of the newly developed CC-C, gait parameters were compared using three-dimensional gait analysis in a patient with chronic stroke–induced hemiplegia using SP-C and CC-C. The use of CC-C may improve gait parameters in patients with stroke-induced hemiplegia, and GRF values may differ with SP-C and CC-C use.

Stroke-induced hemiplegic symptoms reportedly exhibit characteristic spatiotemporal gait parameters [24–27]. In the present case, differences were observed in stride length and single-stance time between the paretic and nonparetic sides (Table 2), consistent with characteristics of previous reports. The bilateral step length, stride length, and cadence were higher in the CC-C than in the SP-C condition, suggesting a direct increase in gait velocity. Among temporal parameters, the single-stance time increased in the CC-C condition compared with that in the SP-C condition, whereas the preswing time decreased. The symmetry of temporal parameters reportedly affects gait velocity [28]. In the present case, the single-stance time in the CC-C condition increased and approached the single-stance time on the nonparetic side (i.e., the paretic swing phase), which may have contributed to gait velocity. These differences in temporal parameters also indicate that the relative time at each level during gait was different, which can be interpreted as different gait patterns under the two conditions based on the concept of a generalized motor program [29]. Both variation in spatiotemporal parameters and improvement in gait pattern may have contributed to the faster gait velocity in the CC-C condition.

Furthermore, the improvement in gait pattern can be explained by ROM and COM trajectories during gait. As shown in Table 3, in the SP-C condition, the mean maximum right lateral flexion angle of the trunk during walking was −0.7°, while the mean left lateral flexion angle was 5.3°, indicating that the trunk was tilted to the left (paretic side) throughout the gait cycle. In contrast, in the CC-C condition, the mean maximum right lateral flexion angle of the trunk was 1.5°, whereas the mean maximum left lateral flexion angle was 2.1°, indicating a symmetrical gait. Considering that trunk and pelvic movements during gait have been reported to affect the lateral displacement of COM and gait rhythm [6, 30] in the CC-C condition compared with the SP-C condition, the symmetry of trunk motion in this case was considered to have led to an increase in the bilateral single-stance time and lateral COM shift distance and a decrease in the left–right difference in the lateral COM trajectory. In the vertical direction, the COM trajectory of the single-stance time increased in the CC-C condition and approached the trajectory of single-stance time on the nonparetic side. Since the trajectory of the vertical COM shift during a single stance is shorter in patients with stroke-induced hemiplegia than in healthy people [31], the use of CC-C could have possibly reduced this characteristic abnormal gait. The hip and knee-joint angles increased in the CC-C condition in ROM of the lower limb on the paretic side during gait. Recently, the terminal stance hindlimb-extension angle and trailing-limb angle have been reported to influence gait and propulsion in stroke-induced hemiplegia [32, 33]. This hypothesis was confirmed in the present case, and in the CC-C condition, the increased hip-extension angle on the paretic side could have created a propulsive force toward the swing phase. In addition, the increased maximum hip and knee joint-flexion angles on the paretic side that occurred during the swing phase as well as the increased paretic step and stride lengths also seemed to support the hypothesis of an increased propulsion force.

Next, the researchers considered how differences in gait patterns were affected by differences in canes. A comparison of the GRF applied to the cane during walking showed no differences between the two conditions in the vertical component. Contrastingly, in the sagittal plane, the braking force tended to increase in the SP-C condition, whereas the propulsion force increased in the CC-C condition. Patients with stroke-induced hemiplegia reportedly have a decreased propulsion force on the paretic side of the lower limb, which is associated with gait velocity [34–36]. Although reports analyzing the effect of a cane on propulsion during gait are scarce, Kuan et al. [14] reported that cane usage increased the spatial parameters of step length and stride length in stroke-induced hemiplegia and that these were effects of push-off by the cane. The CC-C used in this case has a resilient quality unique to carbon: the shape of the cane foot is suitable for push-off. Therefore, CC-C use could result in a propulsion force by the cane.

Chen et al. [37] reported that a propulsion force was formed on the nonparetic lower limb, and the cane provided support and braking function; this mechanical component analysis during cane gait was performed on 20 patients with stroke-induced hemiplegia. In contrast, this report shows the possibility of supplementing propulsion with a cane as well as with a nonparetic lower limb. The medial component was the highest in the SP-C condition. Further, in the SP-C condition, a left–right difference was observed in the trunk ROM and COM trajectory in the frontal plane of gait. This was considered a condition in which lateral stability support using a cane was necessary. CC-C also has a larger base of support because the ground-contact portion is a surface rather than a point. Additionally, the rounded shape of the grip may eliminate the need for excessive lateral control of the cane. The extension angle of the nonparetic wrist (i.e., cane side) was reduced in the CC-C condition. In the present case, a difference was observed in the ROM of the upper limb on the paretic side during walking (Table 2). Upper limb motion during gait is closely related to many factors, including velocity and posture [38, 39] as well as the reaction force from the ground [40]. In this case, the difference in ROM was considered to be caused by the confounding effect of multiple factors, including velocity, posture, and use of a cane by the nonparetic side upper limb.

Table 4 presents the results of the practical balance evaluation tests and usability evaluations that were not limited to a straight-line gait. The CC-C appears to be useful in ensuring ADL independence. The results of the 10 m maximum walking test and TUG test were comparable with those of the SP-C condition, and usability evaluation showed that the CC-C condition was comparable with the SP-C condition.

A limitation of this study is that it did not bilaterally measure the GRF applied to the lower extremities. A force plate was employed for the cane developed in this study to compare it with a conventional cane. This allowed us to verify the difference in GRF values generated by the cane; however, the researchers were unable to examine the effect on the paretic leg, which was grounded simultaneously as the cane. The gait pattern and velocity improved when using CC-C. The propulsion force of the paretic leg is related to gait disorder [33, 36]; it is possible that CC-C had a positive effect on the propulsion force of the paretic leg in this case as well. The researchers believe that measuring the GRF generated by the lower limb simultaneously with the cane will clarify the mechanism of improvement in gait disturbances and the different effects of cane usage. In the future, the researchers aim to study cases in which CC-C is applicable. The usefulness of a cane in stroke-induced hemiplegia may depend on the patient's gait velocity [41, 42]. As the patient in this case tended to have a relatively high gait velocity of 1.05 m/s with a conventional cane (SP-C), studying cases with different speeds and the effects of the intervention earlier is necessary.

In conclusion, this study examined the effect of CC-C use in a patient with chronic stroke–induced hemiplegia. CC-C use improved the patient's gait pattern and velocity. Compared with SP-C, CC-C also provided more propulsion force at the expense of limiting the braking and medial reaction forces. Thus, changing to an appropriate cane may enhance the gait function in patients with stroke-induced hemiplegia. Although canes have traditionally been used for support and stability, CC-C could serve as a new therapeutic intervention as a cane that provides propulsion.

## Figures and Tables

**Table 1:** Demographic characteristics of the patient.

Age (years)	41
Sex	Male
Height (m)	1.75
Weight (kg)	76
Diagnosis	Cardiogenic cerebral infarction
Time after onset (years)	3
Medical history	None
Brunnstrom Recovery Stage	II–III–IV
Stroke Impairment Assessment Set: motor	1-1A-4-3-0
Stroke Impairment Assessment Set: sensory	2-2-3-3
Stroke Impairment Assessment Set: total	37
Functional Independence Measure: total (motor, cognitive)	116 (83, 33)
Mini-Mental State Examination Score-Japanese	30

**Table 2:** Spatiotemporal gait characteristics.

	Single-point cane	Carbon-curved cane	*p* value
Gait velocity (m/s)	1.05 ± 0.05	1.18 ± 0.03	< 0.01
Paretic step length (m)	0.62 ± 0.02	0.65 ± 0.03	0.01
Nonparetic step length (m)	0.65 ± 0.02	0.67 ± 0.03	< 0.01
Stride length (m)	1.27 ± 0.03	1.33 ± 0.04	< 0.01
Cadence (steps/min)	99.55 ± 3.92	106.37 ± 1.91	0.01
Step width (m)	0.29 ± 0.02	0.30 ± 0.02	0.34
Loading response time (%)	22.08 ± 1.67	20.95 ± 1.02	0.09
Single-stance time (%)	23.54 ± 1.18	27.19 ± 0.53	< 0.01
Preswing time (%)	23.68 ± 0.68	22.29 ± 1.16	0.01
Swing time (%)	30.70 ± 1.68	29.57 ± 1.08	0.11

**Table 3:** Maximum range of motion and center of mass trajectory while walking.

	Single-point cane	Carbon-curved cane	*p* value
*Trunk maximum range of motion (°)*			
Right/left flex	−0.7 ± 0.8/5.3 ± 0.9	1.5 ± 0.7/2.1 ± 0.7	< 0.01/< 0.01

*Paretic maximum range of motion (°) (flexion/extension)*			
Shoulder	20.9 ± 2.7/−3.3 ± 2.2	17.4 ± 2.5/−6.6 ± 2.7	0.01/0.01
Elbow	72.9 ± 4.4/−70.1 ± 3.6	65.2 ± 3.6/−61.4 ± 5.1	0.01/< 0.01
Wrist	32.2 ± 1.1/−30.8 ± 0.4	33.1 ± 0.7/−30.3 ± 0.4	0.07/0.06
Hip	24.8 ± 1.0/9.5 ± 1.1	27.2 ± 1.0/11.6 ± 1.5	< 0.01/0.01
Knee	54.1 ± 1.9/−2.5 ± 1.8	58.1 ± 1.9/−4.2 ± 2.2	< 0.01/0.02
Ankle	−2.1 ± 1.0/12.4 ± 0.9	−2.8 ± 1.2/13.2 ± 0.5	0.27/0.07

*Nonparetic maximum range of motion (°) (flexion/extension)*			
Shoulder	11.6 ± 2.8/14.8 ± 1.8	12.9 ± 2.4/13.4 ± 2.6	0.27/0.23
Elbow	28.5 ± 4.1/-7.8 ± 2.3	31.8 ± 3.3/−7.4 ± 1.4	0.09/0.51
Wrist	2.3 ± 0.7/15.1 ± 2.0	1.8 ± 0.5/8.2 ± 1.1	0.12/< 0.01
Hip	31.4 ± 1.7/14.2 ± 1.3	32.0 ± 1.5/14.9 ± 0.9	0.20/0.19
Knee	63.2 ± 1.4/−7.9 ± 1.6	63.9 ± 1.1/−7.8 ± 1.1	0.20/0.62
Ankle	4.8 ± 1.2/16.1 ± 1.1	5.3 ± 0.9/16.7 ± 0.6	0.18/0.11

*Center of mass trajectory (mm)*			

*Swing phase*			
Vertical	21.7 ± 2.9	24.1 ± 3.6	0.23
Lateral	22.3 ± 3.4	24.6 ± 1.6	0.09

*Single-stance phase*			
Vertical	17.1 ± 3.4	23.6 ± 3.6	< 0.01
Lateral	16.6 ± 2.5	22.1 ± 1.6	< 0.01

**Table 4:** Balance test and usability scale scores.

	Single-point cane	Carbon-curved cane
10 m maximum walking test		
Speed (s)	7.25	6.82
Steps (step)	17	16
**Timed up and go** (s)	12.43	10.58
**System Usability Scale** (score)	45	95
Quebec User Evaluation of Satisfaction with Assistive Technology		
Satisfaction (score)	2.50	4.00
Service (score)	2.75	4.25
Whole (score)	2.63	4.13

## Data Availability

The data that support the findings of this study are available on request from the corresponding author. The data are not publicly available due to privacy or ethical restrictions.
